# Lung hemorrhage and brain stroke following fatal viper (cerastes cerastes) bite

**DOI:** 10.11604/pamj.2017.26.99.11727

**Published:** 2017-02-24

**Authors:** Salah Bellasri, Hicham Janah

**Affiliations:** 1 Medical Imaging Department, Military Hospital, University Mohammed V, Rabat, Morocco; 2 Respiratory Department, Military Hospital, University Cadi Ayyad, 40010 Marrakech, Morocco

**Keywords:** Envonimation, lung hemorrhage, acute stroke

## Image in medicine

A 46-year-old with a history of mild hypertension was working outside when a viper bit him on his right hand. The patient arrived at the nearest medical facility about 30 minutes after and received polyvalent antivenom serum, bite site care. He was taken to the emergency department (ED) by ambulance. At ED of our center, 2 hours after bite, the patient was unconscious, his blood pressure was initially unobtainable, and his cardiac monitor revealed a rate of 147/min. Breathlessness, hemoptysis and rales were heard in the lung bases. Neurological examination revealed right hemiplegia, a right facial droop, and dysarthria. A Foley catheter was placed and returned a hemorrhagic fluid. A blood samples examinations showed: a hematocrit was 45%, prothrombine (PT) greater than 120 seconds, HGB 8.7 g/dl, platelets 30 x10^3^ /mm^3^. Blood oxygen saturation after oxygen facemask was low, and the patient was intubated, using in endotracheal tube and was placed on a ventilator. 6 hours following admission, after the patient was somewhat stabilized, a noncontrast computerized tomographic (CT) scan of the head demonstrated multiples stroke with hemorrhagic infarction left middle cerebral artery territory (a, b). A chest CT scan revealed an area of alveolar condensation reaching the superior right lobe and Fowlers segment related to hemorrhagic intra alveolar bleeding (c, d). The morning following admission, the patient was found to be hypoxic. His systolic blood pressure decreased. Later on second day, the patient became flaccid with nonreactive pupils and he was pronounced dead later the same day.

**Figure 1:**
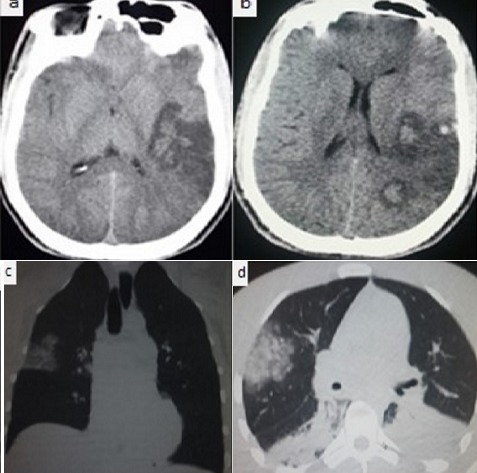
A computed tomography: (a, b) axial images of brain: show area of stroke in the left middle artery territory with hemorrhagic infarction; (c,d) chest coronal and axial images, in lung window: demonstrate an area of intra alveolar bleeding, involving the superior right pulmonary lobe and both the Fowler segment

